# Effectiveness of hepatoprotective drugs for anti-tuberculosis drug-induced hepatotoxicity: a retrospective analysis

**DOI:** 10.1186/s12879-016-2000-6

**Published:** 2016-11-11

**Authors:** Zenya Saito, Yugo Kaneko, Akira Kinoshita, Yusuke Kurita, Kyuto Odashima, Tsugumi Horikiri, Yutaka Yoshii, Aya Seki, Yoshitaka Seki, Hiroshi Takeda, Kazuyoshi Kuwano

**Affiliations:** 1 Division of Respiratory Diseases, Department of Internal Medicine, The Jikei University Daisan Hospital, 4-11-1 Izumihoncho, Komae-shi, Tokyo 201-8601 Japan; 2 Division of Respiratory Diseases, Department of Internal Medicine, The Jikei University School of Medicine, Tokyo, Japan

## Abstract

**Background:**

The effectiveness of hepatoprotective drugs for DIH (drug induced hepatotoxicity) during tuberculosis treatment is not clear. We evaluated the effectiveness of hepatoprotective drugs by comparing the period until the normalization of hepatic enzymes between patients who were prescribed with the hepatoprotective drugs after DIH was occurred and patients who were not prescribed with the hepatoprotective drugs.

**Methods:**

During 2006–2010, 389 patients with active tuberculosis were included in this study. DIH was defined as elevation of peak serum aspartate aminotransferase (AST) and/or alanine aminotransferase (ALT) of more than twice the upper limit of normal (ULN). We divided the patients into the severe (peak serum AST and/or ALT elevation of >5 times the ULN), moderate (peak serum AST and/or ALT elevation of >3 to ≤5 times the ULN), and mild DIH groups (peak serum AST and/or ALT elevation of >2 to ≤3 times the ULN). We compared the average period until the normalization of hepatic enzymes between patient subgroups with and without hepatoprotective drugs (ursodeoxycholic acid: UDCA, stronger neo-minophagen C: SNMC, and glycyrrhizin).

**Results:**

In the severe group, there was no significant difference in the average period until the normalization between subgroups with and without hepatoprotective drugs (21.4 ± 10.8 vs 21.5 ± 11.1 days, *P* = 0.97). In the mild group, the period was longer in the subgroup with hepatoprotective drugs than that without hepatoprotective drugs (15.7 ± 6.2 vs 12.4 ± 7.9 days, *P* = 0.046).

**Conclusion:**

Regardless of the severity, hepatoprotective drugs did not shorten the period until the normalization of hepatic enzymes.

## Background

Tuberculosis (TB) is one of the serious infectious diseases. Isoniazid, rifampicin, and pyrazinamide are the first-line standard-regimen drugs administered for TB, but these anti-TB drugs often bring some major adverse effects such as hepatotoxicity, gastrointestinal and neurological disorders, and skin reactions [1–3]. Especially, drug-induced hepatotoxicity (DIH) is one of the most serious adverse effects ascribed to anti-TB drugs. Larrey reported that hepatotoxicity accounted for more than 7.0 % of all adverse effects [4]. Hepatotoxicity diminishes the effectiveness of anti-TB drugs because it leads to significantly poor adherence, and eventually it can lead to not only treatment failure but also recurrence of TB and drug-resistance [5, 6].

In 2006, the American Thoracic Society (ATS) reported that the frequency of DIH ranged from 5 to 33 %, and the risk factors included age over 35, female sex, alcohol use, preexisting liver damage, the presence of rifampicin in a multidrug treatment regimen, history of viral hepatitis, and use of second-line anti-TB agents [7]. Thereafter, many studies have also shown the relationship between DIH and risk factors, but few have shown the relationship between DIH and hepatoprotective drugs. Although there is no consensus on their effectiveness against DIH, hepatoprotective drugs have often been used empirically in patients with DIH, such as herbal medicine. If hepatoprotective drugs improve hepatic enzymes in patients suffering from DIH, it may be possible to prevent interruption and failure of treatment. Therefore, we evaluated the effectiveness of hepatoprotective drugs by comparing the period until the normalization of hepatic enzymes between patients with and without hepatoprotective drugs.

The purpose of this study was to investigate the effectiveness of several hepatoprotective drugs (ursodeoxycholic acid: UDCA, stronger neo-minophagen C: SNMC, and glycyrrhizin) in patients with hepatic enzyme elevation owing to anti-TB drugs.

### Hepatoprotective drugs

UDCA, SNMC, and glycyrrhizin are often used in Japan as hepatoprotective drugs.

### UDCA

UDCA, a hydrophilic dihydroxylated bile acid, was first identified as a major constituent of the dried bile of the Chinese black bear. Reports on a beneficial effect on serum liver tests in cholestatic disorders were first published in Western literature [8–10]. The structure of UDCA was already elucidated in 1936 by Iwasaki [11], and the effectiveness of UDCA against chronic hepatitis was first reported in 1961 [12, 13]. Since then, many studies have shown the effectiveness of UDCA in chronic hepatitis, and the mechanism of its action has been recognized as (1) replacement/displacement of toxic endogenous bile acids, (2) cytoprotective effects on hepatocytes and bile duct epithelial cells, (3) immunomodulatory effects, and (4) stimulation of bile secretion by hepatocytes and bile duct epithelial cells [14–16].

### SNMC

SNMC, an intravenous drug, contains glycyrrhizin as a principal ingredient as well as glycine and L-cysteine. It has been used to treat chronic hepatitis for over 30 years in Japan and has been shown to be effective in preventing hepatocellular carcinoma development in patients with chronic hepatitis C; however, its underlying mechanisms of action remain to be elucidated [17]. In Japan, SNMC has been used for treating allergic diseases since 1948 and was also used for treating chronic liver disease. Suzuki et al. reported that serum levels of AST, ALT, and γ-GTP can be significantly reduced by SNMC [18]. In Japan, the use of SNMC has been approved for managing liver function abnormalities in chronic liver disease since 1979.

### Glycyrrhizin

Glycyrrhizin, a conjugate of one molecule of glycyrrhetinic acid with two molecules of glucuronic acid, is extracted from the roots of the *Glycyrrhiza glabra* plant has been used for treating chronic hepatitis for over 20 years [19]. It has been used as an anti-allergic agent in traditional Chinese medicine and as a food additive in beverages and licorice because of its sweet taste [20]. In 1946, Revers reported on the anti-ulcer effect of licorice [21]. Since then, glycyrrhizin has been used in Europe as an anti-ulcer drug for several years. In 1977, Suzuki et al. reported that the plasma transaminase activity in a group of patients with chronic active liver disease treated with glycyrrhizin improved significantly compared to the placebo-treated group [18]. However, the mechanism of action of glycyrrhizin remains unknown.

### Association between hepatotoxicity and the isoniazid metabolic pathway

An association between hepatotoxicity and the isoniazid metabolic pathway has been reported in recent studies [22–27]. According to these studies, the predominant metabolic pathway of isoniazid metabolism is acetylation by the hepatic enzyme N-acetyltransferase 2. As the acetylation rate in humans is genetically determined, humans can be categorized into slow and rapid acetylators. In rapid acetylators, isoniazid is acetylated into acetylisoniazid and then excreted as diacetylhydrazine via acetylhydrazine. In slow acetylators, however, isoniazid is directly hydrolyzed into isonicotinic acid and hydrazine. These studies have concluded that this hydrazine is the most likely cause of isoniazid-induced hepatotoxicity. The mechanism of rifampicin- and pyrazinamide-induced hepatotoxicity remains unclear, but it is known that rifampicin increases isoniazid hydrolase activity and production of the hepatotoxic metabolite of isoniazid in slow acetylators [28].

## Methods

### Study population and treatment

The study population included all patients with active TB diagnosed by smear, culture, or polymerase chain reaction (PCR) analysis of sputum specimens, gastric washing, or bronchoalveolar lavage between January 2006 and December 2010 at the Jikei University Daisan Hospital. Clinical data were obtained retrospectively from medical records. Blood tests were preformed at least once a week during the initial 2 months of TB therapy. All patients received first-line standard-regimen drugs such as daily isoniazid, rifampicin, and ethambutol (HRE regimen) or isoniazid, rifampicin, ethambutol, and pyrazinamide (HREZ regimen). According to the Japanese Society for TB, the dosages of anti-TB drugs were 5 mg/kg/day (maximum 300 mg/day) of isoniazid, 10 mg/kg/day (maximum 600 mg/day) of rifampicin, 25 mg/kg/day (maximum 750 mg/day) of ethambutol, and 25 mg/kg/day (maximum 1.5 g/day) of pyrazinamide [29]. This study was approved by the institutional ethical committee at The Jikei University School of Medicine.

### Definition of anti-TB DIH

**Fig. 1:**
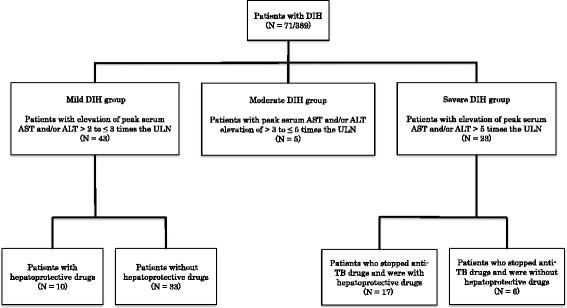
Patient selection flow chart. Of the 389 patients diagnosed as active TB, 71 was identified to develop DIH, with 23 patients assigned to the severe, five patients to the moderate, and 43 patients to the mild DIH group

According to the Japanese Society of TB, anti-TB DIH was evaluated by serum aspartate aminotransferase (AST), alanine aminotransferase (ALT), and total bilirubin values. All patients in the present study have an increase of serum AST and ALT, but few patients have an increase of total bilirubin. As a result, we evaluated the serum AST and ALT as markers of hepatotoxicity. The normal range of serum AST and ALT was defined as less than 30 U/L by referring the Japanese Society of Laboratory Medicine. Also, DIH was defined as elevation of peak serum and/or ALT > 2 times the upper limit of normal (ULN) from the beginning of treatment. All patients with DIH were divided into three groups as shown in Fig. 1. First, the patients with DIH were divided into the severe, mild, and moderate DIH groups by the severity of their hepatotoxicity. The severe DIH group was defined as elevation of peak serum AST and/or ALT > 5 times the ULN. The moderate group was defined as elevation of peak AST and/or ALT > 3 to ≤ 5 times the ULN. The mild DIH group was defined as elevation of peak serum AST and/or ALT > 2 to ≤ 3 times the ULN. Next, these groups were divided into subgroups of patients with and without hepatoprotective drugs. The following patients were excluded: (1) patients with a history of liver disease such as viral hepatitis or other liver diseases, and (2) patients with a serum AST and/or ALT > the ULN before anti-TB treatment.

### Management of hepatotoxicity

When DIH occurred, anti-TB drugs were continued in all patients within the moderate and mild DIH groups, but these drugs were stopped immediately in all patients within the severe DIH group. Then, the decision of whether to use hepatoprotective drugs in all of the patients with hepatotoxicity was based on the judgment of the attending physicians.

### Comparison factor between patients with and without hepatoprotective drugs

The purpose was to investigate retrospectively the effectiveness of hepatoprotective drugs in patients with hepatic enzyme elevation owing to anti-TB drugs. We retrospectively obtained the clinical data from medical records, and compared the average period until the normalization of the hepatic enzymes between patients with and without hepatoprotective drugs. We evaluated statistically the effectiveness of hepatoprotective drugs by comparing the differences of this period.

### Statistical analysis

The Mann-Whitney *U* test was used to compare the differences in average values, and the chi-square test was used to compare paired proportions. A *P* value < 0.05 indicated statistical significance for all analyses. All statistical analyses were performed with SPSS software, version 20.0 (SPSS Inc., Chicago, IL, USA).

## Results and discussion

### Baseline characteristics of the patients with DIH

Of the 389 patients diagnosed as having active TB, 71 (18 %) were identified as developing DIH, of whom 23 patients were assigned to the severe, 5 patients to the moderate, and 43 patients to the mild DIH groups. However, it was impossible to include the moderate group in the analysis because the number of applicable patients was too low. In the severe DIH group, 17 patients received hepatoprotective drugs and 6 patients did not. In the mild DIH group, 10 patients received hepatoprotective drugs and 33 patients did not. There were no significant differences in sex and age between the subgroups with and without hepatoprotective drugs as shown in Tables 1 and 2. Peak AST/ALT levels tended to be higher in each group with hepatoprotective drugs, but the differences were not significant.

**Table 1:** Baseline characteristics of the 23 patients with severe drug-induced hepatotoxicity

	Patients who stopped anti-TB drugs and received hepatoprotective drugs (*N* = 17)	Patients who stopped anti-TB drugs but did not receive hepatoprotective drugs (*N* = 6)	*P* value
Sex			1.00
Male	12	4	
Female	5	2	
Age, years	64 ± 20	58 ± 22	0.58
Peak AST (IU/dl)	477 ± 404	278 ± 156	0.31
Peak ALT (IU/dl)	362 ± 188	262 ± 132	0.55

Data are expressed as number or means ± standard error

*TB* tuberculosis, *AST* aspartate aminotransferase, *ALT* alanine aminotransferase

**Table 2:** Baseline characteristics of the 43 patients with mild drug-induced hepatotoxicity

	Patients with hepatoprotective drugs (*N* = 10)	Patients without hepatoprotective drug (*N* = 33)	*P* value
Sex			0.80
Male	7	24	
Female	3	9	
Age (years)	57 ± 21	55 ± 23	0.80
Peak AST (IU/dl)	66 ± 19	58 ± 12	0.20
Peak ALT (IU/dl)	72 ± 21	54 ± 21	0.058

Data are expressed as number or means ± standard error

*AST* aspartate aminotransferase, *ALT* alanine aminotransferase

### Average period until the normalization of hepatic enzymes

In the severe DIH group, as shown in Table 3, there was no significant difference in the average period until the normalization of the hepatic enzymes between the patient subgroups with and without hepatoprotective drugs (21.4 ± 10.8 vs 21.5 ± 11.1 days, *P* = 0.97). As shown in Table 4, the average period until the normalization in the mild group was longer in the subgroup with hepatoprotective drugs than in the subgroup without (15.7 ± 6.2 vs 12.4 ± 7.9 days, *P* = 0.046).

**Table 3:** Comparison of the average period until normalization of hepatic enzymes in the severe DIH and its HREZ regimen group

	Patients who have stopped anti-TB drugs and with hepatoprotective drugs	Patients who have stopped anti-TB drugs and without hepatoprotective drugs	*P* value
severe DIH group	21 ± 10 days (*N* = 17)	21 ± 11 days (*N* = 6)	0.97
HREZ group	21 ± 8 days (*N* = 11)	24 ± 10 days (*N* = 5)	0.64

Data are expressed as means ± standard error

*Abbreviations*: *H* isoniazid, *R* rifampicin, *E* ethambutol, *Z* pyrazinamide

**Table 4:** Comparison of the average period until normalization of hepatic enzymes in the mild DIH and its HREZ regimen group

	Patients with hepatoprotective drugs	Patients without hepatoprotective drugs	*P* value
Mild DIH group	15 ± 6 days (*N* = 10)	12 ± 7 days (*N* = 33)	0.046
HREZ group	16 ± 7 days (*N* = 9)	13 ± 9 days (*N* = 26)	0.061

Data are expressed as means ± standard error

*Abbreviations*: *H* isoniazid, *R* rifampicin, *E* ethambutol, *Z* pyrazinamide

### Average period until the normalization of hepatic enzymes in the HREZ regimen group

All patients received first-line standard-regimen drugs such as HRE or HREZ regimen. The patients of HREZ regimen and not HRE regimen have a higher risk of hepatotoxicity than the patients of HRE regimen and not HREZ regimen because pyrazinamide have hepatotoxicity as side effect. Among the patients treated with the HREZ regimen in the severe DIH group, as shown in Table 3, there was no significant difference in the average period until the normalization of hepatic enzymes between those with and without hepatoprotective drugs (21 ± 8 vs 24 ± 10 days, *P* = 0.64). Of the patients treated with the HREZ regimen in the mild DIH group, as shown in Table 4, there also was no significant difference in the average period until the normalization of hepatic enzymes between those with and without hepatoprotective drugs (16 ± 7 vs 13 ± 9 days, *P* = 0.061).

### Flow of the design and definition decisions

The present study showed that hepatoprotective drugs did not shorten the period until the normalization of hepatic enzymes regardless of the severity of the hepatotoxicity caused by anti-TB drugs. Miyazawa et al. reported in 2003 that glycyrrhizin, one of the hepatoprotective drugs, was not effective in patients with elevated hepatic enzymes owing to anti-TB drugs [30]. To our knowledge, however, no study reported subsequently on the relationship between anti-TB DIH and hepatoprotective drugs. From this point of view, the present study has great value in terms of investigating the effectiveness of hepatoprotective drugs including UDCA and SNMC. Also, these hepaprotective drugs have never been evaluated the possibility of prophylactic effect. We believe that it is worth discussing this possibility as another potential prospective study.

Many risk factors are associated with DIH. In particular, several studies have reported a positive association between viral hepatitis and DIH [31–33]. In the present study, we excluded those patients with viral hepatitis and other liver disease to eliminate the possibility of DIH other than that of liver enzyme elevation occurring during TB treatment.

As per the hepatotoxicity definition, both the ATS and the British Thoracic Society (BTS) have recommended that potentially hepatotoxic medications should be halted if the serum ALT exceeds 5 times the ULN (with or without symptoms) or 3 times the ULN with jaundice and/or hepatitis symptoms. Furthermore, regular monitoring of liver function is recommended when the serum ALT is > 2 times the ULN. Otherwise, the Japanese Society for TB recommends that medications should be stopped if serum AST and/or ALT exceed either 5 times the ULN (with or without symptoms) or 3 times the ULN with symptoms. Referring to these recommendations, we defined DIH as an elevation of peak serum AST and/or ALT of > 2 times the ULN to detect more patients with DIH [34]. DIH occurred in 18 % of patients who underwent anti-TB treatment in the present study. Although a wide definition of DIH was used in the present study in comparison with that of the ATS and the BTS, the percent of patients with DIH was similar to that in several previous studies [35, 36]. The ATS has also recommended that anti-TB drugs should be resumed after the serum ALT level drops to < 2 times the ULN. In present study, we resumed anti-TB drugs in all patients after both serum AST and ALT returned to normal levels.

### Limitations of the present study

The present study has some limitations. First, because this is a retrospective study, the medication period and dosage of hepatoprotective drugs were not unified. Some patients were treated with two or more hepatoprotective drugs at the same time. Therefore, we could not evaluate the effectiveness of each hepatoprotective drugs individually. To our knowledge, the action of mechanism for Glycyrrhizin, SNMC, and UDCA were not clarified completely. We believe that it is necessary to examine the efficacy of each drug separately in a future prospective study. Second, we excluded the moderate DIH group because it comprised only 5 applicable patients. We also defined DIH as an elevation of peak serum AST and/or ALT > 2 times the ULN to detect more patients with DIH, but only 71 patients fit our criteria. Further investigation via a large prospective study is necessary in the future. Third, UDCA, SNMC, and glycyrrhizin are normally used for viral hepatitis and cholelithiasis, but the use of these hepatoprotective drugs for DIH has never been permitted as health care services provided by health insurance in Japan. This fact may delay investigation into the association between DIH and hepatoprotective drugs. Despite the above limitations, we believe this study has valuable implications for clinical practice.

## Conclusion

In patients with active TB, hepatoprotective drugs did not appear to shorten the period until the normalization of hepatic enzymes, regardless of the severity of the hepatotoxicity caused by anti-TB drugs.

## Abbreviations

ALT: Alanine aminotransferase
AST: Aspartate aminotransferase
ATS: American Thoracic Society
BTS: British Thoracic Society
DIH: Drug-induced hepatotoxicity
HRE: Isoniazid, rifampicin, and ethambutol
HREZ: Isoniazid, rifampicin, ethambutol, and pyrazinamide
PCR: Polymerase chain reaction
SNMC: Stronger neo-minophagen C
TB: Tuberculosis
UDCA: Ursodeoxycholic acid
ULN: Upper limit of normal
